# IKK*β *inhibitor identification: a multi-filter driven novel scaffold

**DOI:** 10.1186/1471-2105-11-S7-S15

**Published:** 2010-10-15

**Authors:** Shanthi Nagarajan, Hyunah Choo, Yong Seo Cho, Kye Jung Shin, Kwang-Seok Oh, Byung Ho Lee, Ae Nim Pae

**Affiliations:** 1 Neuro-Medicine Center, Life/Health Division, Korea Institute of Science and Technology, PO Box 131, Cheongryang, Seoul 130-650, Republic of Korea; 2 School of Science, Korea University of Science and Technology, 52 Eoeun dong, Yuseong-gu, Daejeon 305-333, Republic of Korea; 3 Drug Discovery Division, Korea Research Institute of Chemical Technology, Daejeon, Republic of Korea

## Abstract

**Background:**

Nuclear factor kappa B (NF-κB) is a chief nuclear transcription factor that controls the transcription of various genes; and its activation is tightly controlled by Inhibitor kappa B kinase (IKK). The irregular transcription of NF-κB has been linked to auto-immune disorders, cancer and other diseases. The IKK complex is composed of three units, IKK*α*, IKK*β*, and the regulatory domain NEMO, of which IKK*β *is well understood in the canonical pathway. Therefore, the inhibition of IKK*β *by drugs forms the molecular basis for anti-inflammatory drug research.

**Results:**

The ligand- and structure-based virtual screening (VS) technique has been applied to identify IKK*β *inhibitors from the ChemDiv database with 0.7 million compounds. Initially, a 3D-QSAR pharmacophore model has been deployed to greatly reduce the database size. Subsequently, recursive partitioning (RP) and docking filters were used to screen the pharmacophore hits. Finally, 29 compounds were selected for IKKβ enzyme inhibition assay to identify a novel small molecule inhibitor of IKK*β *protein.

**Conclusions:**

In the present investigation, we have applied various computational models sequentially to virtually screen the ChemDiv database, and identified a small molecule that has an IC_50 _value of 20.3*μ*M. This compound is novel among the known IKK*β *inhibitors. Further optimization of the hit compound can reveal a more potent anti-inflammatory agent.

## Background

Inhibitor kappa-B kinase*β *(IKK*β*) is a serine-threonine protein kinase, which is critically involved in the activation of transcription factor Nuclear Factor kappa B (NF-κB) in response to various inflammatory stimuli [1]. IκB, an inhibitory unit, is responsible for retaining NF-κB in the cytoplasm [2], for the degradation of IκB by phosphorylation, and for ubiquitination to translocate NF-κB into the nucleolus, leading to transcription initiation [3]. IKK*β *plays a crucial role in the way of canonical NF-κB pathway, which phosphorylates IκB protein and thereby translocates NF-κB into the nucleus and initiates pro-inflammatory gene transcription. The canonical NF-κB pathway is well recognized in chronic inflammatory diseases [4] and inhibition of the IKK*β *enzyme by a highly potent inhibitor has remained the primary goal for anti-inflammatory drug discovery.

The IKK complex comprises two catalytic subunits, IKKα and IKKβ, and a regulatory subunit, IKKγ. Although both the catalytic subunits can catalyze the phosphorylation of IκB*α*, the IKK*β *subunit seems to play a dominant role in the canonical pathway. Furthermore, IKK*α *has a crucial role in mediating p52 activation through the 'non-canonical' pathway [5]. IKK*α *can form an alternative complex (without IKK*β *and IKKγ) and its function is required for the development of the lymphoid organ and the maturation of B cells [6]. Termination of the canonical pathway by inhibiting IKK*β *is a potential target in anti-inflammatory drug research.

Recently, the virtual screening (VS) method is playing an increasingly important role in drug discovery. The structure-based method involves docking of small molecules and ranking them based on their score. Every scoring function has its own inherent limitations, and thus, there is a high chance for reporting false positives. In order to minimize the risks of using a structure-based approach, additional filters have been used to enrich the VS scheme. The application of various computational filters in the VS cascade certainly alleviates the difficulties encountered during the initial stages of the drug discovery process. Every model used in the VS scheme has been meticulously validated by test sets that are not included in training the models. In general, the performance of the model is highly dependent on the choice of the ligand that used to train the model.

## Results and discussions

### 3D-QSAR pharmacophore model

**Figure 1:**
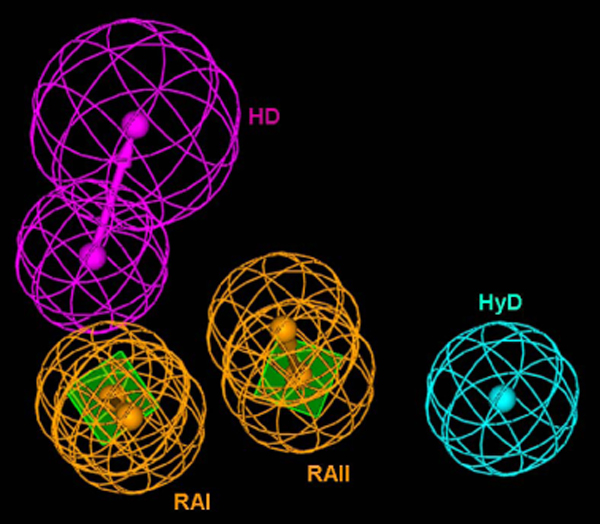
**The Hypogen model composed of two ring aromatic (RAI and RAII), one hydrophobic and one hydrogen bond donor features**.

Among the 10 pharmacophore models generated, model 1 was considered to be the best, because it has the lowest RMSD value (0.89Å) and a high correlation coefficient (r = 0.93) between the experimental and estimated activity data of the training set. The difference between the total and the null hypothesis cost is 40.21. If the difference is 40-60 bits, then there is a 75-90% chance that this model can represent a true correlation of the data. Additionally, the difference between null and fixed costs is more than 50 and the configuration cost is 16.17, which is less than the maximum threshold of 17. Cost analysis has confirmed that the statistical relevance of pharmacophore **1 **being a reliable model in forecasting the activity precisely. Model **1 **has four features, comprising an HD, two RA and an HyD (Fig. 1) and has been rigorously validated by estimating the activity of 136 compounds, whose experimental activity range span four orders of magnitude. The estimated activity is found to be fairly good and the correlation value (***r***) between the experimental and estimated value is 0.77. Detailed information about this pharmacophore is described elsewhere [7].

### Recursive partitioning model

The decision tree developed based on the IKK*β *inhibitors is effective in differentiating between IKK*β *inhibibitors and non-inhibitors rapidly. Moreover, this model exhibits a high level of accuracy of 89.8% and 73.8% for the training and test sets, respectively. Table 1 explains the statistical measures that support this model. The sensitivity of RP models is usually found to be higher than the specificity, with respect to training and test sets. Therefore, this model is effective in precisely classifying inhibitors and non-inhibitors. The precision value can demonstrate the capability of the RP model in predicting active compounds [8]. The observed Kappa values of the training set (0.97) and test set (0.77) indicate that the predictivity of the RP model is not by chance [9]. The Matthews Correlation Coefficient (MCC) has been used to measure the quality of binary classifications. The MCC values are 0.8 and 0.4 with respect to the training and test sets, signify improved prediction over random classification. Based on the satisfactory statistics obtained by this model, we have used the RP model for the virtual screening cascade, in order to classify active and inactive compounds from the large database.

**Table 1:** Statistical analysis of the RP model.

Data set	**Accuracy **^**a **^**(%)**	**Sensitivity **^**b **^**(%)**	**Specificity **^**c **^**(%)**	**Precision **^**d **^**(%)**	**Kappa **^**e**^	**MCC **^**f**^
Training	89.8 (202/225)	92.2 (95/103)	87.7(107/122)	86.3	0.97	0.8
Test	73.8 (62/84)	77(47/61)	65.2 (15/23)	85.4	0.77	0.4

^a^Accuracy = (TP + TN)/(TP +TN + FP + FN); ^b^Sensitivity = TP/(TP + FN); ^c^Specificity = TN/(TN + FP); ^d^Precision = TP/(TP + FP); ^e^Kappa = accuracy-E/1-E, where E = expected agreement = (TN+FN) (TN+FP) (FP+TP) (FN+TP)/(TP+FP+FN+TN)^2^; ^f^MCC = (TP * TN) - (FP * FN)/√(TP + FN) (TP + FP) (TN+ FP) (TN + FN).

#### Decision tree

**Figure 2:**
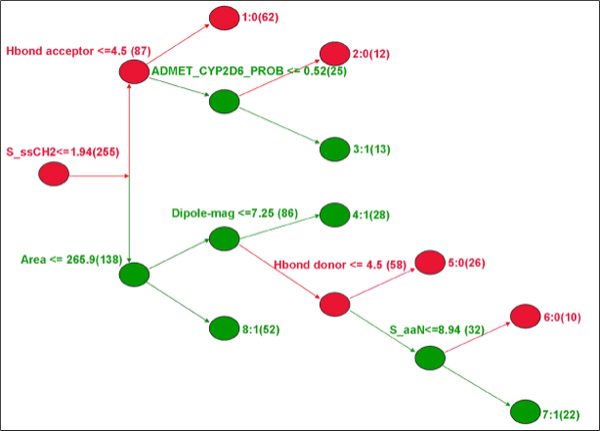
**The RP decision tree showing the chief decisive factors, the red nodes representing the active class and the green nodes representing the inactive class**.

The RP model has been characterized by five branches and eight nodes, and each node contains information on the classification of either 'active' or 'inactive' compounds (Fig. 2). The tree is composed of various descriptors; of these, the chief descriptor belongs to the electrotopological category. It can encode information for both the topological environment of an atom and its electronic interactions with all other atoms in the molecule.

The S_ssCH_2 _is the first decisive factor, which stands for the sum of intrinsic values for the -CH_2_- atom type with two single bonds (SP^3^). The descriptor indicates that generally active compounds have alkyl groups. The second descriptive factor is the hydrogen bond acceptor that represents interaction with the hinge loop. Most of the active compounds have a minimum of four donor features, implying that any one of the acceptor features can have an interaction with the hinge loop donor. Similarly, on of the other decisive descriptors, the hydrogen bond (Hbond) acceptor can also explain the same concept *vice versa*. The other decisive factors are CYP2D6 inhibition, area, dipole-mag, Hbond donor, and S_aaN. An explanation corresponding to each descriptor is provided in Table 2.

**Table 2:** Summary of the descriptors that were found to be useful in decision making in the RP model

Descriptor	Illustration
S_ssCH2	Sum descriptor for carbon with two single bonds.
Hbond Acceptor	Number of hydrogen bond acceptor
ADMET_CYP2D6_PROB	Prediction of CYP2D6 inhibition.
Area	Molecular surface area.
Dipole-mag	The strength and orientation behaviour of a molecule in an electrostatic field.
Hbond donor	Number of hydrogen bond donor
S_aaN	Sum descriptor for nitrogen with two aromatic bonds.

### Virtual Screening

**Figure 3:**
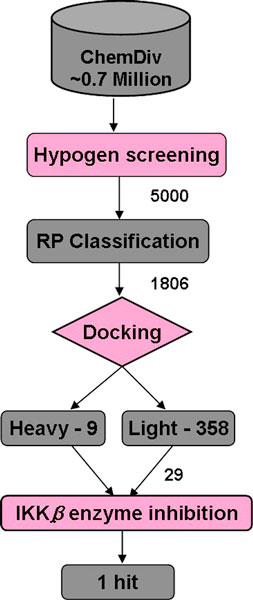
**Schematic representation of the virtual screening cascade**.

By using the above mentioned models, we have been able to filter the ChemDiv database, that has approximately 0.7 million compounds (Fig. 3). We have used a Hypogen pharmacophore model as a primary filter. The database search retrieved 15,110 hits and the top scoring 5,000 compounds with reasonable fit-values, which are in the range 7.61-9.17 have been considered for further filtering. Following the pharmacophore search, the RP classification model has been applied to 5000 compounds, of which 1806 compounds are classified as IKK*β *inhibitors. In the VS cascade, the final filter is molecular docking. All 1,806 compounds are subjected to heavy and light constrained docking and as a result, 6 and 358 hit compounds were reported, respectively. Finally, the top scoring (based on f-scores) 31 compounds from both docking methods have been selected. Of these, only 29 compounds available from suppliers were subjected to *in vitro *screening.

### Hit analysis

**Figure 4:**
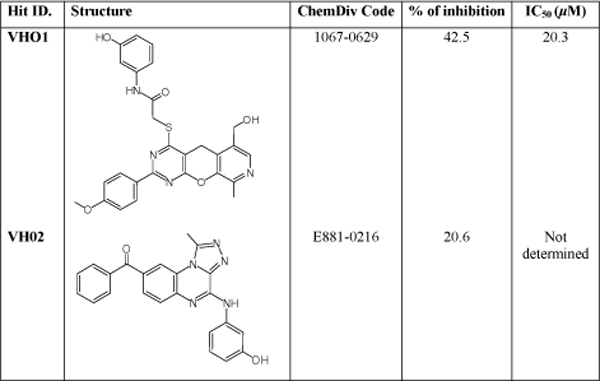
**The compounds having an inhibition rate > 20% at 10*μ*M and their IC_50 _values**.

**Figure 5:**
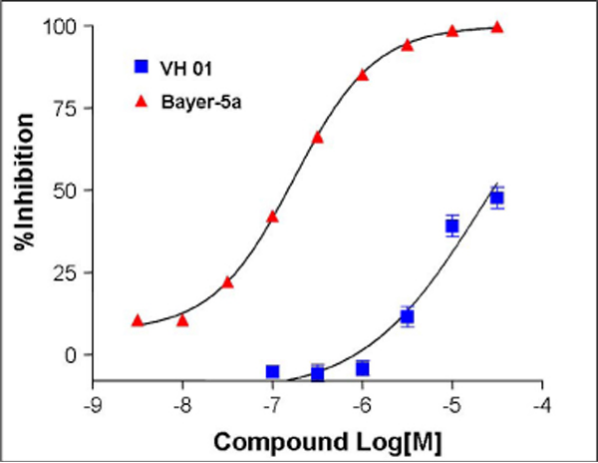
**TR-FRET analysis of IKK*β *phosphorylation inhibition by VH01 and Bayer-5a, a known compound used as a positive control**.

The IKK*β *enzyme inhibition screening of 29 compounds revealed that two compounds have an inhibition effect of more than 20% at 10*μ*M concentration (Fig. 4). The first compound, with 42.5% of inhibition, was found to have an IC_50 _value at 20.3 *μ*M (Fig. 5). The positive control, Bayer-5a has been measured to have an IC_50 _value of 0.17 *μ*M, which is 6.96 fold higher than that reported by Murata *et al. *[10] and could be due to differences in assay conditions. Based on the Bayer-5a screening result, it is expected that the hit compounds will be more potent in recombinant human IKK*β *inhibition assays.

**Figure 6:**
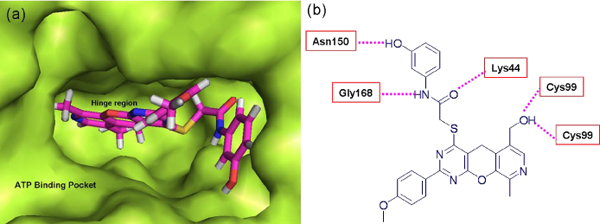
**(a) Surface view of the ATP binding site and the docked conformation of the hit compound**. (b) 2 D representation of the hydrogen bond formed between hit compound and the adjacent residues.

The hit molecule VH01 is based on a pyran moiety that makes five Hbond interactions at the ATP binding pocket (Fig. 6a), two Hbonds (Acceptor and donor) with the hinge region Cys99, and establishes three other bonds between various functional groups of lead molecules and residues such as Lys44, Gly168 and Asn150 (Fig. 6b). The molecule can be stabilized well in the pocket and therefore, has a high docking score of -22.60. The reported hit molecule is specifically derived from a light constraint method, because heavy constraints force the conformation of any molecule to interact with the hinge region. Therefore, the docking score falls as these compounds can now make ideal interactions with the hinge region; however, they fail to inhibit IKK*β *in real time. Hence, we have proposed the light constraint approach, that can be applied to locate molecules in the deep buried binding pocket as the heavy constraint method can only produce unrealistic hits. Moreover, our previously reported screening also supports the light constraint method [11].

**Figure 7:**
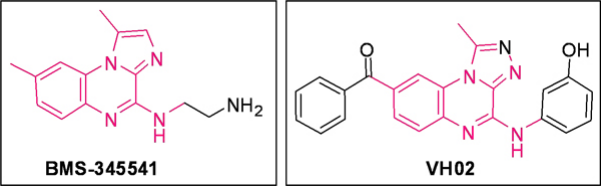
**Similarity between the BMS compound and the VH02 compound, the pink regions depicting the core scaffold similarity**.

The VH02 compound has a low inhibition effect of 20.6% at 10 *μ*M concentration, due to which it was not considered further for IC_50 _calculation. However, similarity searching reveals that the compound has a high degree of similarity with the imidazoquinoxaline derivative BMS-345541 (Fig. 7), that can potently inhibit IKK*β *(IC_50 _= 0.3 *μ*M) and has 13-fold selectivity over IKK*α*.

The chemical similarity between the VH02 and BMS-34541 provides a basic intuition for the chemical modification of this hit compound. The benzaldehyde moiety of VH02 can be replaced by tiny hydrophobic moieties, whereas, the phenol moiety can be replaced by pyrrole, that can maintain the same distance constraint for nitrogen as that of the BMS compound, to facilitate hydrogen bond formation between the NH group of the ligand and the receptor.

## Conclusion

We have developed a filter-driven scaffold model and applied it for the virtual screening of IKK*β *inhibitors. Sequential filtering of the database can reduce the false positive rate to a large extent at each stage. The first two models are generated by means of using the known inhibitor information and the third model is a structure-based approach. At the initial level of screening, IKK*β *inhibitor-like compounds are retained, and allowed to pass on to the structure-based filter. Docking of several compounds simultaneously to the IKK*β *active site revealed the set of compounds that are stable at the ATP binding pocket. In general, identification of lead molecules using a computational modeling approach often relies on approximation and has limited accuracy. Therefore, the VS hits have been validated further by subjecting them to *in vitro *studies.

The VS approach reported 367 hits; and among these compounds, only 29 have been selected based on encouraging scores, diversity, and commercial availability for the IKK*β *inhibition assay. Of the 29 compounds tested, we have identified one hit (VH01) with IC_50 _20.3 *μ*M. Despite this inhibition value, this compound is found to be structurally novel among reported IKK*β *inhibitors. There are series of similar compounds patented by Zhuravel *et al. *[12], which interestingly, also seem to exhibit antitumor activity. Hideshima *et al. *[13] have previously explained the use of a small molecule inhibitors of IKK*β *and its role in inhibiting the haematological cancer, multiple myeloma. Accordingly, we will focus our attention on the anti-cancer point of view with the identified hit compound. Further optimization of VH01 can lead us to discover more potent compounds that can act as anti-inflammatory as well as anti-cancer agents, and this work currently underway. Although the VH02 compound has not been found to be very potent, its similarity to BMS-345541 has suggested that the screening system could bring out the core features required to be present in the IKK*β *inhibitor. Moreover, the VS cascade is not based on serendipity, as it has proven its efficiency in identifying IKK*β *inhibitors.

## Methods

### Pharmacophore model generation

The pharmacophore hypothesis modeling was performed using the Catalyst 4.11 (Accelrys, 9685 Scranton Road, San Diego, Calif. 92121) package. A total of 159 compounds collected from the literature [4,14-17], was made into a library. Subsequently, the library was divided into training and test sets composed of 23 and 136 compounds, respectively. From each scaffold category, a few representative compounds were chosen based on diverse substituents with a wide range of activity data. The 3D-QSAR pharmacophore model known as Hypogen was generated based on 23 IKK*β *inhibitors, whose activity data ranges from 3 nM ≤ IC_50 _≤ 50000 nM. Detailed information about the pharmacophore can be found elsewhere [7]. The training set compounds were broadly classified into four groups: those with an activity range < 100 nM were classified as highly active (+++); an activity range between > 100 nM to < 1 μM were defined as active (++); compounds with an activity range of > 1 μM to < 10 μM were defined as moderately active (+); and, the compounds having an IC_50 _value > 10 μM were classified as inactive (-). The same grouping strategy was applied to the test set compounds also. Excluding the training set compounds, the remaining compounds were used as an internal test set to measure the efficiency of the pharmacophore model; no outliers were removed to achieve unrealistic higher correlation values. These compounds also covered a wide range of activity of 4 nM ≤ IC_50 _≤ 50000 nM.

For every training set compound, all possible conformers were enumerated and a spreadsheet was prepared with the corresponding activity data and conformers. Additional specifications were made to select desired features, such as hydrogen bond donors, hydrogen bond acceptors, hydrophobes and aromatic rings. The spreadsheet was input to the Catalyst program and in a reasonable time-frame, 10 hypotheses were generated. The best pharmacophore model was selected based on highest correlation, lowest RMSD and the most significant cost values.

### Decision tree generation

The RP method of the Cerius2 program was used to generate a decision tree. RP is a classification structure-activity relation (CSAR) method that enables rapid classification of large databases, is non-parametric and captures nonlinear relationships automatically performed based on the Classification and Regression Trees (CART) algorithm [18]. The working principle behind the RP is assembling a set of descriptors, converting them into a data object to reflect the presence or absence of useful features, assembling the data objects into vectors and then into a matrix. Finally, the matrix is divided into two daughter sets, based on the presence/absence of certain useful features. The process is repeated until each member of the matrix has been designated to a terminal node based on the presence/absence of specified features. The RP model is found to be sensitive to the descriptors used, and diversity of the data sets can radically change the property of the decision tree. The method is applicable to structurally unique compounds with activity data to uncover sub-structural rules that govern the biological activity [19]. The RP classification tree is often of great interest to visualize the distribution of potencies at the node and to see how a split at a node divides the potencies at two daughter nodes. This method has been repeatedly used by researchers of bioinformatics and chemoinformatics, either to classify genes or to differentiate active and inactive compounds [20-23]. However, the limitation of the RP method is its inability to extrapolate beyond the range of observed responses. The main objective of incorporating the RP method in the virtual screening process is to rapidly classify unknown compounds based on a small number of readily interpretable descriptors; therefore, for screening compounds.

The recursive partition decision tree model was constructed using a QSAR module of Cerius2 version 4.10.17[24]. The splits were scored using the Gini Impurity scoring function, which minimizes the impurity of the nodes resulting from the split. The tree was set to prune backward through a moderate pruning process, to avoid over splitting. Every node should contain 1% of the samples to qualify for further splits. The knot value was limited to a threshold of 20 per variable and maximum tree depth was set to 10. The best RP tree was generated with these parameters.

### Training and test sets of the RP model

A total of 225 compounds collected from the literature [10,15,16,25-29] were classified into two categories: the active class (0), which includes the compounds having an activity range below or equal to 500 nM; and the inactive class (1), which covers the activity range of more than 500 nM in the IKK*β *enzyme inhibition assay. Two-dimensional and three-dimensional descriptors of Cerius2 were used for the RP tree generation. The descriptors were optimized by means of removing those with constant values and 95% of the zero values, while some of the descriptors were deleted on the basis of the correlation threshold > = 0.9. Totally, 37 descriptors were retained in the RP study that comprised 31 two-dimensional and 6 three-dimensional descriptors (Table 3). In the RP study, we defined the activity class (0 or 1) column as a dependent variable (Y) and the descriptors used as independent variables (X).

**Table 3:** Molecular descriptors used for recursive partition model development

Descriptor class		Descriptors
Structural	2D	MW, Rotlbonds, Hbond acceptor, Hbond Donor
Electrotopological (E-state key)	2D	S_sCH3, S_ssCH2, S_aaCH, S_sssCH, S_tsC, S_dssC, S_aasC, S_sNH2, S_ssNH, S_tN, S_aaN, S_sssN, S_sOH, S_dO, S_ssO, S_sBr
Electronic	2D/3D	Apol, Dipole-mag
Spatial	3D	RadOfGyration, Area, Vm, Density, PMI
Thermodynamic	2D	AlogP98, logP, Molref, Fh2O, Foct
ADME	2D	ADMET_Abos, ADMET_Solu, ADMET_Hepatox, ADMET_CYP2D6, ADMET_PPB

A total of 84 compounds were used as an external test set compounds, collected from a different set of published articles, with none of the compounds or similar scaffolds included in the training set. External test set compounds have been reported by two groups [4,30]. The first set of compounds are derivatives of the imidazothienopyrazine core [30], with a series of compounds having imidazoquinoxaline [15] synthesized by same group included in training the model. Another set of compounds reported by Chiristoper *et al. *[4], was synthesized based on the benzimidazole core to specifically inhibit IKK*μ*, but instead inhibited IKK*β*. The external test sets were combined to serve as an independent test set to asses the generality of the model. Dependent and independent variables were calculated as explained before.

### Docking procedure

The third filter used in the VS scheme was molecular docking. To date, there is no crystal structure reported for IKK*β*. Hence, we modeled the protein based on four other closely related kinase proteins, based on the procedure of homology modeling detailed elsewhere [11]. The templates (Table 4) are human calmodulin-dependent protein kinase (PDB: 2JC6), rat calmodulin-dependent protein kinase (PDB: 1A06), kinase and ubiquitin-associated domains of MARK3/Par-1 (PDB: 2QNJ) and Proetin kinase A-fivefold mutant model of Rho-kinase (PDB: 2GNG).

**Table 4:** The templates used to model IKK*β *protein

PDB	Identity	Protein function	Resolution in Å
2JC6	31%	Crystal Structure of Human Calmodulin-dependent protein kinase 1 D.	2.30
1A06	29%	Calmodulin-dependent protein kinase from rat.	2.50
2QNJ	32%	Kinase and Ubiquitin-associated domains of MARK3/Par-1	2.70
2GNG	29%	Protein kinase A - fivefold mutant model of Rho-kinase	1.87

The FlexX docking program [31] was employed in the structure-based VS. Prior to docking, hydrogen atoms were added to the protein, and it was minimized using the steepest descent algorithm for about 500 steps. The amino acids Phe26, Val29, Ala42, Lys44, Met65, Val74, Ala76, Glu97, Tyr98, Cys99, Lys106, Val152, and Gly168 and the surrounding residues within the distance range of 6.5 Å were defined as active site. FlexX uses an incremental construction algorithm to place flexible ligands into a fully specified active site, while its empirical scoring function estimates the binding free energy based on physicochemical properties. The FlexX-Pharm [32] module was used to define the constraints and direct the FlexX docking of several compounds into the specified active site simultaneously. FlexX-Pharm ensures that an interaction is formed between the specified interacting group in the active site and the ligand in a valid docking solution. There are many research groups, who have successfully employed constraints in structure-based VS to increase the enrichment factor [33-36] of active compounds. As we know, most of the ATP competing kinase protein inhibitors make two or three hydrogen bonding interactions with the hinge region [37-39]. Hence, we applied hydrogen bonding as constraints to select compounds that can possibly make hinge interactions. In the docking simulation, two different sets of constraints were applied; namely, 'heavy' and 'light'. The heavy constraint method is very strict in choosing compounds. According to this method, compounds forming three hydrogen bonds with the hinge region (Acceptor (Glu97)-Donor (Cys99)-Acceptor (Cys99)) were alone reported as hits. In the light constraint method, the middle donor interaction is essential and at least one acceptor hydrogen bonding interaction is essential. A maximum of 30 conformers were retained for each compound, passing the constraints criteria. In our previous work [40], we demonstrated that the f-scoring function was good enough to discriminate IKK*β *inhibitors from decoys and so, the same scoring function has been applied in this VS scheme.

### *In vitro *analysis: IKK*β *enzyme inhibition assay

IKK*β*-TR-FRET reactions for the search of IKK*β *inhibitors were carried out based upon the suggestions of the IMAP-TR-FRET system (MDS Analytical Technologies, Sunnyvale, Calif., USA). IKK*β *kinase reactions were performed in a reaction buffer (10 mM Tris-HCl, pH 7.2, 10 mM MgCl_2_, 0.05% NaN_3_), containing 1 mM DTT and 0.01% Tween-20 (Sigma-Aldrich Co., St. Louis, Mo., USA) to help stabilize the enzyme. The reactions were done at room temperature for 2 h in white standard 384 plates (3572, Corning Life Sciences, Lowell, Mass., USA), using 0.5 μg/ml IKK*β *(Millipore Co., Billerica, Mass., USA), 1 μM I_K_Bα-derived substrate (5FAM-GRHDSGLDSMK-NH2; R7574, MDS Analytical Technologies), and 3 μM ATP (Sigma-Aldrich Co.) unless otherwise noted. The total reaction volumes were 20 μl and 10 μM, and compounds were preincubated with the IKK*β *enzyme for 10 min before the substrate and ATP were added. For the TR-FRET reaction, 60 μl of the detection mixture (1:600 dilution of IMAP binding reagent and 1:400 dilution of Terbium donor supplied by MDS Analytical Technologies) were added 15 h before reading the plate. The energy transfer signal was measured in a multilabel counter with a TR-FRET option (Victor II, PerkinElmer Oy, Turku, Finland). The counter setting was 340 nm excitation, 100 μs delay, and dual-emission collection for 200 μs at 495 and 520 nm. The energy transfer signal data were used to calculate the percentage inhibition and IC_50 _values. To monitor the assay system and to compare the hit compounds, Bayer compound[10] (Bayer -5a) was used as a positive control.

## List of abbreviations used

3D-QSAR: 3D-Quantitative Structure Activity Relationship; VS: Virtual Screening; IKK: Inhibitor kappa B Kinase; NF-κB: Nuclear Factor kappa B; MCC: Matthews correlation coefficient; RP: Recursive Partitioning; HD: Hydrogen bond donor; RA: Ring aromatic; HyD: Hydrophobic

## Competing interests

The authors declare that they have no competing interests.
